# Chronic small intestinal helminth infection perturbs bile acid homeostasis and disrupts bile acid signaling in the murine small intestine

**DOI:** 10.3389/fpara.2023.1214136

**Published:** 2023-07-06

**Authors:** Jenna M. Lane, Tara P. Brosschot, Dominique M. Gatti, Courtney M. Gauthier, Katherine M. Lawrence, Victoria Pluzhnikova, Lisa A. Reynolds

**Affiliations:** Department of Biochemistry and Microbiology, University of Victoria, Victoria, BC, Canada

**Keywords:** helminths, Bile acids, FXR, Immunomodulation, small intestine, Metabolites

## Abstract

Intestinal helminths have evolved an abundance of immunomodulatory mechanisms to ensure long-lived infections in mammalian hosts. To manipulate mammalian immune responses helminths can directly produce immunomodulatory molecules, but helminth infection can also elicit functional changes in the intestinal microbiome which can impact immune functioning. Here we examined how bile acids (BA)s, a group of host-produced, microbiota-modified immunomodulatory metabolites, were altered in abundance and composition during a murine small intestinal helminth infection. We found that murine helminth infection resulted in consistently reduced concentrations of specific taurine-conjugated primary BAs (T-α-MCA and T-CDCA) in the small intestinal luminal contents of mice. BA transporters facilitate the uptake of BAs from the small intestinal lumen, allowing BAs to engage with nuclear BA receptors, and helminth infected mice showed reduced expression of genes encoding basal BA transporters in the small intestine. Finally, we report that there is reduced signaling through the nuclear BA receptor FXR in both the proximal small intestine and ileum of mice during small intestinal helminth infection. Together, our data reveal disruptions to BA homeostasis and signaling in the small intestine during helminth infection. As BAs are known to impact many aspects of mucosal physiology and immunity, examining the functional consequences of BA disruptions during helminth infection will be an important avenue for future research.

## Graphical Abstract

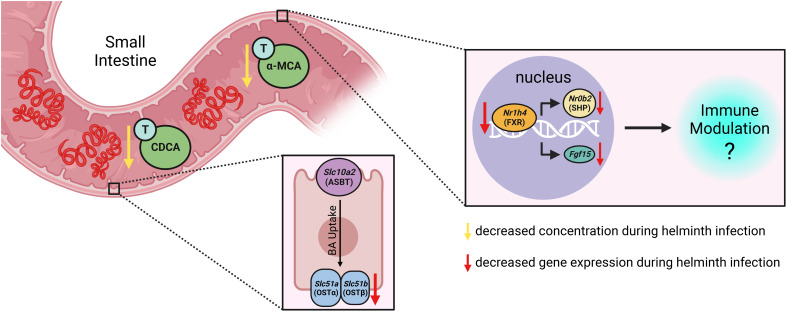

## 1 Introduction

Soil-transmitted helminths are potent manipulators of the mammalian host immune system, and do so by dampening inflammation and inducing regulatory immune pathways in order to establish chronic infections within the gastrointestinal tract (Varyani et al., 2017). Identifying the molecules which mediate helminth-driven immunomodulation is of importance for helminth-infected people and livestock animals, but in addition, may have therapeutic applications for immune-driven inflammatory disorders including inflammatory bowel diseases (Ryan et al., 2020). The majority of efforts to date have concentrated on characterizing molecules directly produced by helminths in their excretory-secretory (ES) material, which has been shown to contain protein, small RNA, and metabolite components each with demonstrated immunomodulatory effects (Yeshi et al., 2022).

As well as ES material produced directly by helminths influencing immune functioning, there are profound changes to the structure and functional capabilities of the bacterial microbiota during helminth infection (Peachey et al., 2017; Llinás-Caballero and Caraballo, 2022). Together, this results in the helminth-infected gut harbouring a distinct concoction of potentially immunomodulatory molecules which differs to that present during homeostasis (Brosschot and Reynolds, 2018). Metabolites within this ‘helminth-modified’ intestinal environment have been shown to impact immune cell functions (Brosschot and Reynolds, 2018), although there is limited progress in both identifying the specific types of metabolites altered during intestinal helminth infection and examining the functional consequences of helminth-modified metabolites *in vivo* (Zaiss et al., 2015; Kennedy et al., 2021; Popple et al., 2021; Shute et al., 2021; Schälter et al., 2022). One group of metabolites that are candidates for contributing to helminth infection-induced immunomodulation are the bile acid (BA)s: a group of molecules deposited in the gut to aid with fat digestion, but which also have increasingly appreciated roles as immune signaling molecules (Chen et al., 2019). BAs can act directly on cell surface and nuclear receptors expressed by mucosal epithelial cells and leukocytes, thereby influencing mucosal immune cell functioning, intestinal motility, and intestinal inflammation (Chen et al., 2019). Two such BA receptors are the nuclear farnesoid X receptor (FXR) and the cell-surface receptor G protein-coupled bile acid receptor 1 (GPBAR1; also known as TGR5), which are expressed in the gut by epithelial cells and various innate immune cells (Chen et al., 2019).

Primary BAs are synthesized by mammals in the liver from cholesterol. Humans produce two primary BAs: cholic acid (CA) and chenodeoxycholic acid (CDCA), whereas mice produce these and an additional three primary BAs: α- and β- muricholic acid (α-/β-MCA) and ursodeoxycholic acid (UDCA) (Li and Dawson, 2019). In both species, primary BAs are conjugated with either glycine or taurine prior to being stored in the gallbladder and subsequently deposited in the duodenum; in mice, taurine conjugation dominates (Li and Dawson, 2019). Conjugation has two main consequences: it allows BAs to form micelles and solubilize dietary lipids, and it reduces the ability of BAs to freely diffuse across intestinal epithelial cell membranes (Dawson and Karpen, 2015). Instead, epithelial transporters are required for the active reuptake of conjugated BAs from the intestinal lumen, which occurs predominately in the ileum (Dawson and Karpen, 2015). Moreover, within the intestinal lumen, conjugated primary BAs can be modified by the bacterial microbiota (to create what are classed as secondary BAs) which has been proposed to reduce their toxicity towards the microbiota and/or to allow for their use by microbial species as an energy source. BAs are first deconjugated by bile salt hydrolases (BSH) encoded by many microbiota species, then they are further metabolized by the microbiota into a plethora of secondary BAs, a small fraction of which are ultimately excreted in feces (Wahlström et al., 2016). Those BAs that are re-absorbed from the intestinal tract recirculate in the blood to the liver, where they can be recycled and ultimately re-deposited in the duodenum as conjugated primary or secondary BAs. This system ensures that under homeostatic conditions the BA pool size is kept relatively constant, with fecal loss being balanced by new liver synthesis.

Here, we show that infection of mice with the small intestinal helminth *Heligmosomoides polygyrus* disrupts BA homeostasis, with a specific reduction in the concentrations of the taurine-conjugated (T-) primary BAs T-α-MCA and T-CDCA in the lumen of the proximal small intestine. Helminth infection further disrupted expression of BA transporter genes in the proximal small intestine and resulted in reduced signaling through the nuclear receptor FXR in both the proximal small intestine and ileum, thereby impacting a BA signaling pathway with established consequences for mucosal immunity (Chen et al., 2019).

## 2 Methods

### 2.1 Mice

All mouse experiments were conducted at the University of Victoria (UVic), approved by the UVic Animal Care Committee, and followed all Canadian Council on Animal Care guidelines (Animal Use Protocol #2021-008). C57BL/6J mice (stock #000664) were purchased from The Jackson Laboratory and either left to acclimatize for a week before being used in experiments, or were subsequently bred in-house and their offspring were used in experiments. Mice from different litters were split evenly between experimental groups, and were 6-11 weeks old at the start of experiments. Mice were housed in groups of 2 - 5 under specific pathogen-free conditions, with *ad libitum* access to food and water. Enrichment included in all cages included crinkle paper, nestlets, and a plastic hut. To avoid time of euthanasia/sample collection influencing experimental results, upon experimental endpoints, one mouse was euthanized from each experimental group sequentially until all euthanasias were complete. Mice were placed in an anaesthetic chamber and exposed to 5% inhaled isoflurane until they reached surgical plane anaesthesia, and then were euthanized by cervical dislocation.

### 2.2 Helminth infection

The life cycle of *H. polygyrus bakeri* was maintained as described previously (Johnston et al., 2015) in C57BL/6J mice. Mice were orally gavaged with 200 third stage *H. polygyrus* larvae. Naive control mice were orally gavaged with water that larvae had been stored in, which had been passed through a 40 μm cell strainer to remove larvae and visually checked under a microscope to ensure water was larvae-free. All mice were euthanized 14 days post-infection/mock infection. Feces were collected from all mice at the point of euthanasia to verify infection status through counting parasite eggs using a McMaster egg counting chamber. As expected, no eggs were detected in the feces of any naive mice, and the feces of all infected mice contained eggs, thus no data points were excluded due to the unexpected presence or lack of parasite eggs.

### 2.3 Sample collection

Blood was collected via cardiac puncture, allowed to coagulate on ice, and spun to obtain sera which was stored at -80°C prior to metabolomic analysis. The proximal small intestine was defined as the first 6 cm following the pyloric sphincter (duodenum and proximal jejunum). The most distal 2 cm of the small intestine was collected for ileal sampling. The contents of the proximal small intestine and ileum were each squeezed out using tweezers. Proximal small intestine contents were flash-frozen with liquid nitrogen and stored at -80°C prior to metabolomic analysis. Proximal small intestinal and ileal tissues were collected into TRIzol™ (Thermo Fisher Scientific, Mississauga, Canada) and stored at -80°C prior to RNA extraction. Entire livers were collected into TRIzol™ and stored at -80°C prior to RNA extraction.

### 2.4 Bile acid quantification

Quantification of BAs was performed at the UVic Genome BC Proteomics Centre, Victoria, Canada, by liquid chromatography (LC)-multiple-reaction monitoring (MRM)/mass spectrometry (MS) using an Agilent 1290 ultra-high-performance liquid chromatography system coupled to a 6495B Agilent QQQ mass spectrometer. The MS instrument was operated in MRM mode with negative-ion detection. A Waters BEH C18 LC column (2.1*150 mm, 1.7 μm) was used, and the mobile phase was 0.01% formic acid in water and 0.01% formic acid in acetonitrile for binary-solvent gradient elution. Specific LC and MS operation parameters were as described previously (Han et al., 2015). Full details can be found in the **Supplementary Material**.

### 2.5 Gene expression analysis

RNA isolations were performed according to the TRIzol™ manufacturer’s instructions. A nanodrop was used to determine RNA concentrations and purity via 260/280 ratios. Agarose gel electrophoresis was then used to confirm RNA integrity through the presence of intact 28S and 18S rRNA bands. RNA concentrations were normalized prior to genomic DNA removal and conversion to cDNA, which were performed with the iScript™ gDNA Clear cDNA Synthesis Kit (Bio-Rad, Mississauga, Canada) according to manufacturer’s instructions. Quantitative PCR was performed using the Applied Biosystems™ PowerUp™ SYBR™ Green Master Mix (Thermo Fisher Scientific, Mississauga, Canada). Each sample was run in triplicate on a Roche LightCycler® 96 Instrument. Relative expression levels of target genes were determined using the ΔΔCq method, with *Gapdh* used as a housekeeping gene because its expression was not affected by *H. polygyrus* infection in any tissue examined (data not shown). Data points were excluded from further analyses if extracted RNA quality was poor.

Primer details and cycling conditions can be found in the **Supplementary Material**.

### 2.6 Statistical analyses

Metabolomics data were first analyzed en masse via a multiple t-test comparison between naive and infected mice for each individual BA species, as well as for broad groups of BAs and total BA levels (consistent standard deviations between samples not assumed, 5% false discovery rate (FDR), two-stage step-up method of Benjamini, Krieger, and Yekutieli). Where discoveries were made (q value ≤ 0.05), individual or groups of BAs were further statistically analyzed as indicated in Figure Legends. For moisture content and gene expression experiments, data were first analyzed for normality using a D’Agostino-Pearson omnibus normality test and a Shapiro-Wilk normality test, then unpaired t-tests or Mann-Whitney tests were used as appropriate and are indicated in Figure Legends. GraphPad Prism version 8 was used for statistical analyses.

## 3 Results and discussion

### 3.1 Small intestinal helminth infection disrupts the bile acid pool

**Figure 1:**
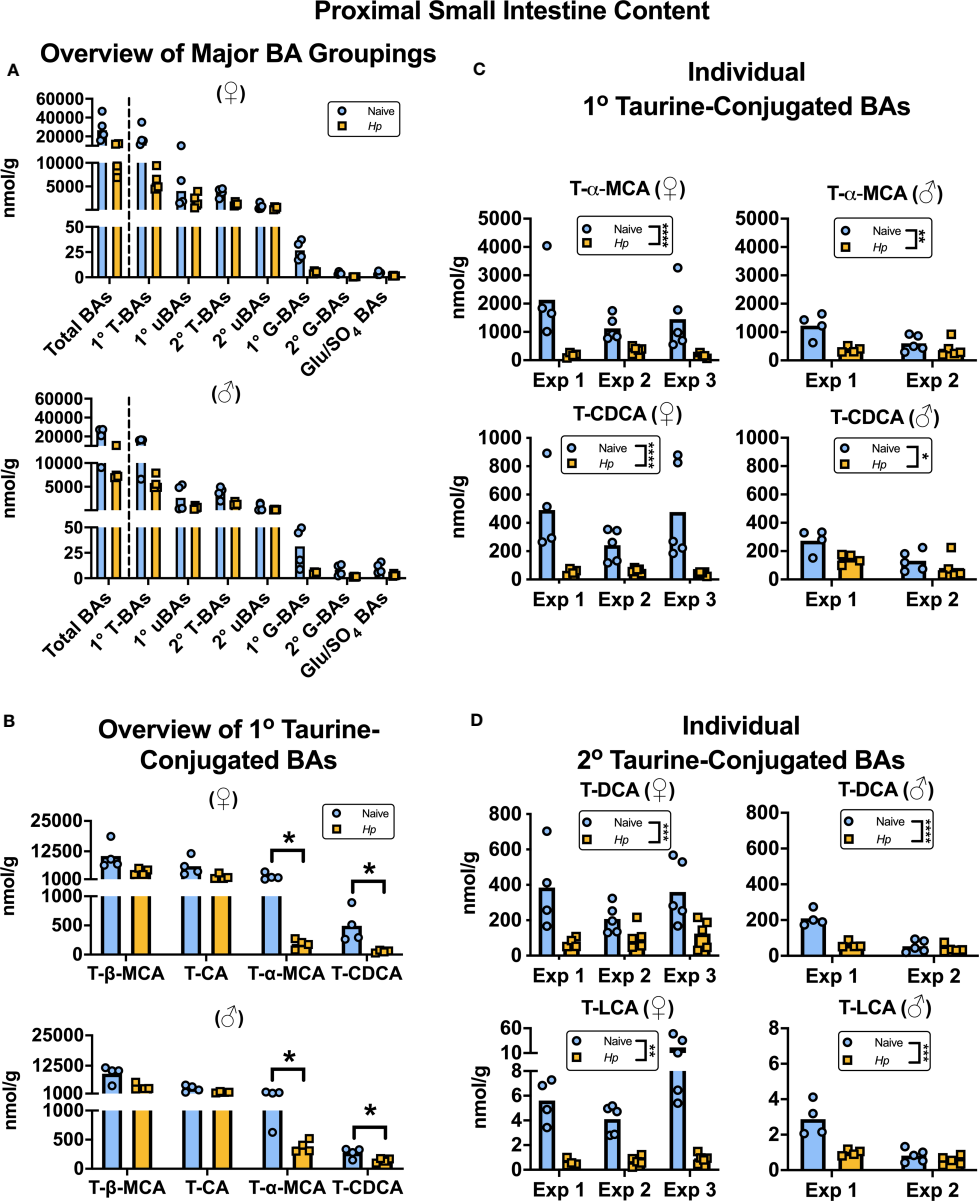
*Heligmosomoides polygyrus* (*Hp*) infection disrupts the bile acid (BA) composition of the proximal small intestinal lumen including a reduction in taurine-conjugated (Τ-)α-MCA and T-CDCA levels. Female (♀) and male (♂) mice were left naive or infected with *Hp*, then BA concentrations were determined and normalized to wet sample weights. **(A)** Total BA concentrations are presented (left side of the dotted line), as well as concentrations of the major groupings of BAs (right side of the dotted line; primary [1°] T-BAs, 1° unconjugated [u] BAs, secondary [2°] T-BAs, 2° uBAs, 1° glycine-conjugated [G-] BAs, 2° G-BAs, and gluconated or sulfated [Glu/SO_4_] BAs). Data shown are from one experiment with an *n*=4 in each experimental group and data from repeat experiments are presented in **Supplemental Figure 1**. **(B)** Concentrations of individual 1° T-BAs. Data shown are from one experiment with an *n*=4 in each experimental group and are representative of the results from three (♀ mice) or two (♂ mice) independent experiments. Although UDCA is a 1° BA in mice, in our assay T-UDCA was indistinguishable from the secondary 2° T-BA T-hyodeoxycholic acid and thus is not included in this graph. A multiple t-test was performed to assess how helminth infection impacted concentrations of all ~80 detectable BA species in our assay, and q values for those BAs presented in this graph are indicated. *q ≤ 0.05. **(C, D)** Concentrations of individual 1° T-BAs **(C)** and individual 2° T-BAs **(D)** across independent experiment (Exp)s, each with an *n*=4-5 in each experimental group. Two-way ANOVA tests were used to assess how Exp and infection status contributed to variation within data sets and the results of the impact of infection status are presented on graphs. *p ≤ 0.05, **p ≤ 0.01, ***p ≤ 0.001, ****p ≤ 0.0001. Each data point represents results from an individual mouse.

To our knowledge, how intestinal helminth infection impacts BA homeostasis has not been previously investigated. We used targeted UPLC-MRM/MS to determine the concentration of ~80 individual BAs in the luminal contents of the murine proximal small intestine during infection with *H. polygyrus*, a strictly enteric helminth which establishes a chronic infection in the proximal small intestine. The size and composition of the BA pool is profoundly influenced by biological sex (Phelps et al., 2019), so we separated female and male mice for all analyses. We calculated BA concentrations normalized to wet sample weights (rather than dry sample weights), as this is more reflective of physiological BA concentrations in the intestinal lumen. When assessing the total BA pool size and the concentrations of the major groups of BAs, we did not find any consistent differences between naive and *H. polygyrus*-infected mice in either sex (**Figure 1A**; **Supplementary Figure 1A**). The most abundant major group of luminal BAs in the proximal small intestine was, as expected (Monte et al., 2009; Li and Dawson, 2019), the primary taurine-conjugated BAs (**Figure 1A**). Within this group, we found that the concentrations of T-α-MCA and T-CDCA were decreased during helminth infection in both female and male mice (**Figure 1B**). This was a consistent finding between repeat experiments using different batches of infectious *H. polygyrus* larvae (**Figure 1C**) which each resulted in robust infections as measured by parasite egg output in mouse feces (**Supplementary Figure 1B**). Concentrations of the secondary taurine-conjugated BAs taurodeoxycholic acid (T-DCA) and taurolithocholic acid (T-LCA) were also consistently decreased during *H. polygyrus*-infection (**Figure 1D**), as were concentrations of the less abundant glycine conjugates glycochenodeoxycholic acid (G-CDCA) and glycohyodeoxycholic acid (G-HDCA) in male and female mice (**Supplementary Figures 1C, D**). Aside from those BA indicated above, we did not find consistent statistical differences in the concentrations of any other BA species during helminth infection when we ran multiple t-test analyses for each of the ~80 detectable BA species, however there were fluctuations in the concentrations of other individual BAs in individual experiments during helminth infection (**Supplementary Data Sheet 1**).

There are likely many contributing factors driving helminth infection-induced disruption to BA concentrations in the proximal small intestine. Firstly, the moisture content of the small intestinal luminal content was increased during *H. polygyrus* infection (**Supplementary Figure 2A**), which was expected due to the “weep and sweep” response during type 2-inducing infections (Varyani et al., 2017). For this reason, we also calculated BA concentrations relative to dry sample weights and found that when we did so, only T-α-MCA, T-CDCA, and T-LCA concentrations were still significantly decreased during helminth infection, and only in female mice (**Supplementary Figures 2B, C**). This suggests that the disruption to BA concentrations during helminth infection is partially, but not completely, due to the influx of fluids that occurs in response to intestinal helminth infections.

We next considered the possibility that host synthesis of BAs was disrupted during helminth infection. To test this, we measured the expression of six murine genes involved in the synthesis and conjugation of BAs (*Cyp7a1*, *Cyp8b1*, *Akr1d1*, *Cyp27a1*, *Cyp2c70* and *Baat*) in the liver (**Supplementary Figure 3**). Of these genes, we only detected a modest downregulation of *Akr1d1* expression during helminth infection, and only in female mice (**Supplementary Figures 3B, C**). This may be a slight contributing factor to the disruption of BA concentrations seen in female mice during *H. polygyrus* infection, as *Akr1d1* knockout mice have a marked reduction in total acid levels including CDCA- and CA- derived BA species (Gathercole et al., 2022).

The bacterial microbiota is known to be disrupted by infection with intestinal helminths (including murine infection with *H. polygyrus*) (Reynolds et al., 2015; Rapin and Harris, 2018), and it is likely that microbiota perturbation also contributes to disruption of BA homeostasis during helminth infection. In the absence of a bacterial microbiota, the BA pool is dominated by conjugated primary BAs, demonstrating the importance of the bacterial microbiota for mediating the deconjugation and further modifications of BAs (Wahlström et al., 2016). Indeed, expression of BSH enzymes which catalyze the deconjugation of BAs is widespread amongst members of the small intestinal bacterial microbiota, with many species encoding multiple distinct BSH enzymes, each with varying degrees of specificity for different conjugated BA substrates (Foley et al., 2019). It is plausible that shifts in the small intestinal bacterial microbiota composition during *H. polygyrus* infection result in increased activity of BSH variants with specificity for T-α-MCA and T-CDCA, explaining the decreased concentrations of T-α-MCA and T-CDCA we observe in the proximal small intestine during helminth infection. Although we did not see a corresponding increase in the concentrations of the unconjugated forms of these BAs in the proximal small intestine contents (α-MCA and CDCA; **Supplementary Data Sheet 1**), it is possible that these metabolites and/or their secondary bile acid derivatives are rapidly removed from this sampling site, perhaps through transport, simple diffusion, or further metabolism by the microbiota. Bacterial microbiota fluctuations between experiments may also contribute to the experiment-to-experiment variability in the influence that *H. polygyrus* has on the overall BA pool size and major groupings that we report (**Supplemental Figure 1A**), as it was recently demonstrated that bacterial microbiota composition shifts during *H. polygyrus* infection vary based on both the starting microbiota composition of mice and on the batch of *H. polygyrus* larvae used for infections (Rapin et al., 2020). It remains to be explored whether *H. polygyrus*, or other intestinal helminth species, have the capacity to directly deconjugate BAs, and if so, if helminth-encoded BSH variants demonstrate substrate specificity for certain types of conjugated BAs.

### 3.2 Reduced expression of small intestinal bile acid transporters during helminth infection

**Figure 2:**
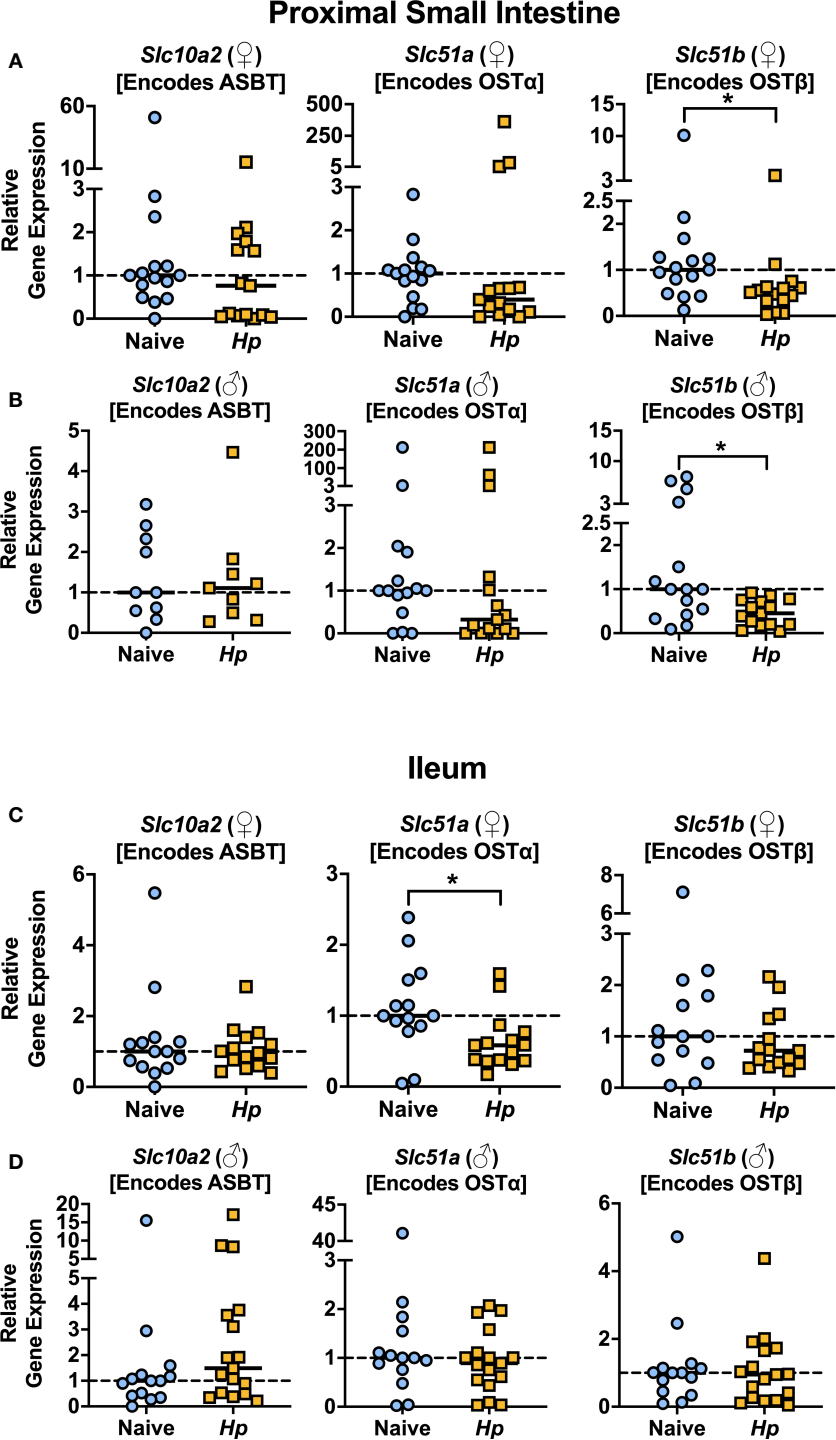
Basolateral bile acid transporter gene expression in the proximal small intestine is reduced during *Heligmosomoides polygyrus* (*Hp*) infection. **(A, B)** Expression levels of the indicated genes for female (♀; **A**) and male (♂; **B**) mice in the proximal small intestine. **(C, D)** Expression levels of the indicated genes for female (♀; **C**) and male (♂; **D**) mice in the ileum. Data shown for *Slc10a2* from ♂ mice in the proximal small intestine are pooled from two independent experiments each with an *n*=4-5 in each experimental group (combined *n*=10 naive mice, *n*=9 infected mice); all other data shown are pooled from three independent experiments each with an *n*=4-6 in each experimental group (combined *n=*15 naive female mice, *n=*15 infected female mice, *n=*15 naive male mice, *n=*15 infected male mice). Statistical comparisons were made using unpaired t-tests if data were parametric, or Mann-Whitney tests if data were non-parametric. *p ≤ 0.05. Each data point represents results from an individual mouse.

With the knowledge that *H. polygyrus* infection resulted in reduced concentrations of T-α-MCA and T-CDCA in the lumen of the proximal small intestine, we next examined whether the capacity for BAs to be reabsorbed from the intestinal lumen was impacted by helminth infection. While unconjugated BAs are able to passively diffuse across the intestinal lumen to enter portal circulation, the major mechanism for the uptake of taurine-conjugated BAs is via active transport into enterocytes through the apical sodium-dependent BA transporter (ASBT), after which BAs are shuttled to the basolateral membrane and effluxed to the blood through the transporter complex organic solute transporter (OST)α-OSTβ (Dawson et al., 2009). We assessed expression levels of the genes encoding these BA transporters in the proximal small intestine, and while we found no significant differences in the expression levels of *Slc10a2* (encodes ASBT) or *Slc51a* (encodes OSTα) during helminth infection, we report a significant, albeit modest, downregulation of one essential component of the basolateral transporter, *Slc51b* (encodes OSTβ), during helminth infection in both female and male mice (**Figures 2A, B**). While downregulation of *Slc51a* (encodes OSTα) during helminth infection did not reach statistical significance in the proximal small intestine, *Slc51a* was modestly but significantly downregulated during helminth infection in the ileum of female mice only (**Figures 2C, D**). Since expression of both OST-α and OST-β can be directly regulated by BAs signaling through BA receptors (Lu et al., 2022), reduced expression of these transporters may be a result of the disrupted luminal BA concentrations we detected during helminth infection, in an attempt to re-establish BA homeostasis.

We next looked in the serum to determine whether the impaired expression of OST-α-OST-β during helminth infection altered concentrations of circulating BA levels during infection but found no consistent disruptions (**Supplementary Figure 4A**), including no differences in circulating levels of any of the individual taurine-conjugated primary BAs in circulation during helminth infection (**Supplementary Data Sheet 1**). It is important to note that the expression of BA transporters is much lower in the proximal small intestine than in the ileum (Dawson et al., 2009) (**Supplemental Figure 4B**), and the ileum is known to be the main site of BA intestinal uptake into circulation (Dawson et al., 2009). That we only detected modest differences in BA transporter gene expression during *H. polygyrus* infection, especially in the ileum, might explain why we do not detect major differences in BA concentrations in circulation during helminth infection. Nevertheless, that we detected reduced BA transporter gene expression during helminth infection was suggestive of disrupted BA signaling, and so we next examined how signaling through BA receptors in the small intestine was impacted during helminth infection.

### 3.3 Helminth infection reduces signaling through FXR in the small intestine

**Figure 3:**
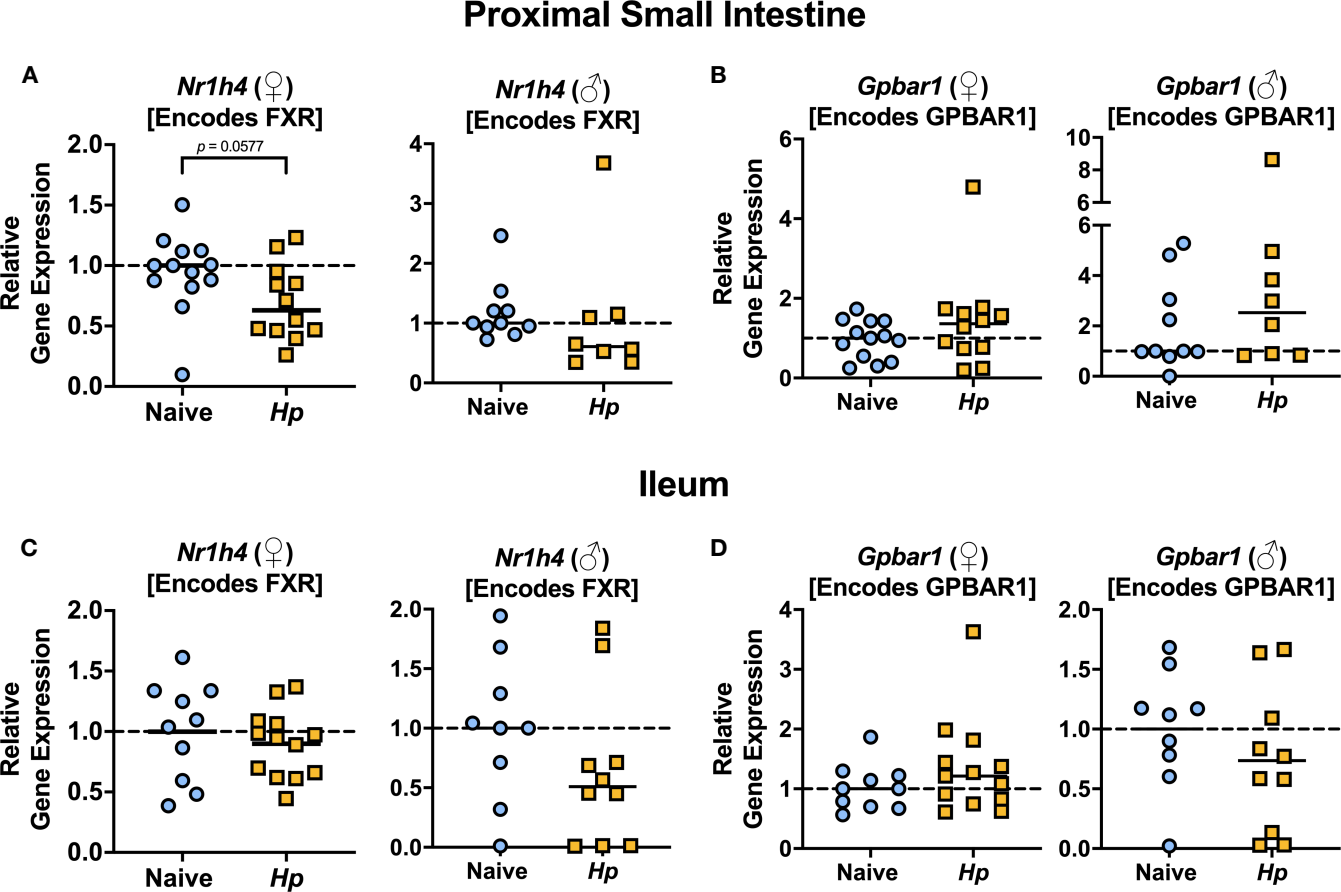
*Heligmosomoides polygyrus* (*Hp*) infection does not significantly alter expression of genes encoding BA receptors FXR or GPBAR1 in the small intestine. **(A, B)**. Gene expression levels of *Nr1h4* **(A)** and *Gpbar1* **(B)** in the proximal small intestine. **(C, D)**. Gene expression levels of *Nr1h4* **(C)** and *Gpbar1* **(D)** in the ileum. Data shown for female (♀) mice are pooled from three independent experiments each with an *n*=2-5 in each experimental group (combined *n*=13 naive mice, *n*=12 infected mice for proximal small intestine samples, and *n*=10 naive mice, *n*=13 infected mice for ileal samples) and data shown for male (♂) mice are pooled from two independent experiments each with an *n*=4-6 in each experimental group (combined *n*=10 for naive mice, *n*=8 for infected mice for proximal small intestine samples, and *n*=9 for naïve mice, *n*=10 for infected mice for ileal samples). Statistical comparisons were made using unpaired t-tests if data were parametric, or Mann-Whitney tests if data were non-parametric. P value was stated if under 0.1. Each data point represents results from an individual mouse.

**Figure 4:**
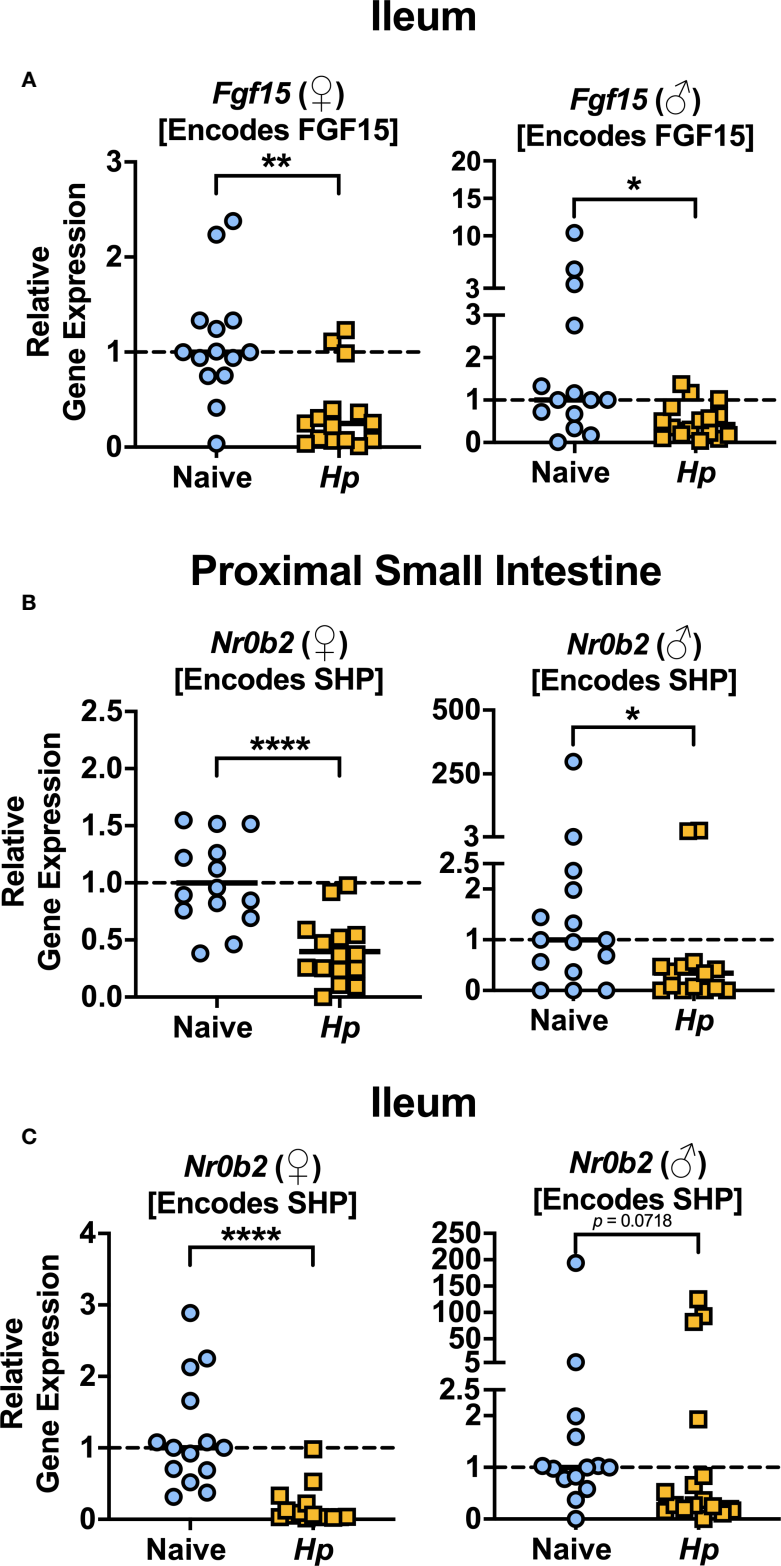
*Heligmosomoides polygyrus* (*Hp*) infection reduces signaling through FXR in the small intestine. **(A)** Gene expression levels of *Fgf15* in the ileum were determined by quantitative PCR. **(B, C)** Gene expression levels of *Nr0b2* in the proximal small intestine **(B)** and ileum **(C)** were determined quantitative PCR. Data shown are pooled from three independent experiments each with an *n*=3-6 in each experimental group (combined *n*=14 naive female (♀), *n*=15 infected female, n=15 naive male (♂), *n*=14 infected male proximal small intestine samples and *n*=14 naive female, *n*=15 infected female, *n*=14 naive male and *n*=16 infected male ileal samples. Statistical comparisons were made using unpaired t-tests if data were parametric, or Mann-Whitney tests if data were non-parametric. *p ≤ 0.05, **p ≤ 0.01, ****p ≤ 0.0001, and p value was stated if under 0.1. Each data point represents results from an individual mouse.

The most well-characterized BA signaling receptors to date are the nuclear FXR and the cell-surface GPBAR1 (Ticho et al., 2019). FXR and GPBAR1 are both highly expressed by many intestinal cells, such as intestinal epithelial and endothelial cells, as well as by innate immune cells including dendritic cells, monocytes/macrophages, NK and NKT cells, and GPBAR1 specifically is also expressed by intestinal muscle and neuronal cells (Biagioli et al., 2021). We set out to determine whether intestinal helminth infection impacted the expression of genes encoding these receptors, and/or if signaling through these receptors was impacted by helminth infection in female and male mice. We did not detect any significant differences in expression of the genes encoding the receptors FXR (*Nr1h4*) or GPBAR1 (*Gpbar1*) themselves during helminth infection (**Figure 3**). T-CDCA and T-α-MCA, the two conjugated BAs that we found to be consistently and significantly decreased in concentration during *H. polygyrus* infection, are potent agonists and antagonists of FXR, respectively (Xiang et al., 2021). To determine the overall impact of helminth infection on signaling through FXR, we examined expression of genes known to be transcribed as a result of FXR activation: *Fgf15* (encodes FGF15) and *Nr0b2* (encodes SHP) (Chen et al., 2019). Detection of *Fgf15* in the proximal small intestine was inconsistent due to low gene expression (data not shown), which is consistent with previous reports (Inagaki et al., 2005). However, we were able to quantify levels of *Fgf15* expression in the ileum and found reduced expression levels during *H. polygyrus* infection in both female and male mice (**Figure 4A**). Further, expression levels of *Nr0b2* were significantly reduced in the proximal small intestine of both female and male mice during helminth infection (**Figure 4B**). In the ileum expression of *Nr0b2* in female mice was also reduced during helminth infection, with a trend for reduced *Nr0b2* gene expression during helminth infection in male mice that did not reach statistical significance (**Figure 4C**). This decreased expression of key genes known to be transcribed downstream of FXR activation strongly indicates that FXR activation is downregulated in both the proximal small intestine (the site of *H. polygyrus* colonization) and in the ileum (the major site for BA transport across the intestinal epithelium, signaling, and interaction with immune cells). Reduced FXR activation during helminth infection may be a consequence of the reduced luminal concentrations of the FXR agonist T-CDCA in the small intestine we report during helminth infection, as T-CDCA is a potent FXR activator (Xiang et al., 2021).

The consequences of an altered intestinal bile acid composition and reduced FXR activation in the small intestine during helminth infection will be important areas for future investigation. For example: could reduced FXR activation impact the chronicity of helminth infection? While the detection of bile has been implicated as a habitat selection cue for helminths such as *H. polygyrus* along the intestinal tract (Sukhdeo and Croll, 1981), and indeed microbiota depletion (which impacts the BA pool) results in altered *H. polygyrus* distribution along the intestinal tract (Moyat et al., 2022) to our knowledge, whether modified signaling through BA receptors impacts the host immune response during helminth has not yet been explored. It has been reported that FXR activation can inhibit intestinal IL-1β, IL-6, and TNF-α, and GPBAR1 activation can attenuate IL-12 and TNF-α production by dendritic cells (Fiorucci et al., 2021). There is evidence that some of these cytokines can reduce immunity to intestinal helminth infection: for example, IL-6 and IL-12 have each been shown to limit Th2 responses associated with *H. polygyrus* expulsion (Smith and Maizels, 2014; Coomes et al., 2015), and further, dietary delivery of BAs which activate FXR result in elevated intestinal type 2 responses which accelerated expulsion of *Nippostrongylus brasiliensis* (Arifuzzaman et al., 2022). Although we did not directly assess levels of GPBAR1 activation during helminth infection, we did find that levels of taurolithocholic acid (T-LCA), a potent agonist of GPBAR1 (Maruyama et al., 2002), were significantly reduced in the content of the proximal small intestine during *H. polygyrus* infection, which could in turn lead to reduced GPBAR1 activation and an increase in IL-12 production. It remains to be determined whether the reduced FXR signaling we observe in the small intestine during *H. polygyrus* infection is promoting helminth chronicity, perhaps through elevated IL-6/IL-12 levels or other mechanisms limiting the efficacy of type 2 responses.

The intestinal epithelium is comprised of diverse cell types including enterocytes, goblet cells, tuft cells, Paneth cells, M cells, and enteroendocrine cells. Several of these cell types have been shown to respond to and/or contribute to the host response against helminth infection (Baska and Norbury, 2022), and it will be important for future work to consider the impact of disrupted bile acid concentrations on the functioning of each of these cell types during helminth infection. Small intestinal tuft cells, for example, have a critical role in sensing helminth infection and stimulating type two innate lymphoid cell (ILC2) expansion through the production of IL-25, thus initiating a type 2 immune response which is critical for helminth expulsion (Faniyi et al., 2020). Tuft cells are particularly abundant in the mucosa of the gallbladder and extrahepatic bile ducts, and the abundance of these biliary tuft cells was recently shown to be negatively regulated by dietary administration of CA (O’Leary et al., 2022). While the abundance of small intestinal tuft cells was not impacted by CA-feeding in this report (O’Leary et al., 2022), it will be worth future work investigating how the helminth-modulated BA pool impacts tuft cell populations, given the importance of this cell type for anti-helminth immunity. Beyond helminth infection impacting signaling through FXR, helminth infection-induced shifts in the primary and secondary BA pool may impact signaling through other cell surface and nuclear receptors known to be responsive to BAs [recently reviewed in (Biagioli and Fiorucci, 2021)], with further potential consequences for intestinal physiology, motility and immunity. It will be important for future research to explore the broader consequences of disrupted BA homeostasis during helminth infection, for example, to determine whether BA disruptions contribute to the altered susceptibility to various enteric microbial infections that have been reported during intestinal helminth infections (Chen et al., 2005; Osborne et al., 2014; Reese et al., 2014; Su et al., 2014; Reynolds et al., 2017; Brosschot et al., 2021; Ingle et al., 2021).

## Data availability statement

The original contributions presented in the study are included in the article/**Supplementary Material**. Further inquiries can be directed to the corresponding author.

## Ethics statement

The animal study was reviewed and approved by University of Victoria's Animal Care Committee.

## Author contributions

JL and TB designed experiments, performed experiments, analyzed and interpreted data, and wrote the manuscript. DG, CG, KL, and VP performed experiments and interpreted data. LR conceived the study, designed experiments, performed experiments, analyzed and interpreted data, and wrote the manuscript. All authors contributed to the article and approved the submitted version.
