# Supplementary material for: Chronic small intestinal helminth infection perturbs bile acid homeostasis and disrupts bile acid signaling in the murine small intestine
Source: Front Parasitol. 2023 Jul 6;2:1214136. doi: 10.3389/fpara.2023.1214136 (PMC11731828; doi:10.3389/fpara.2023.1214136)
Supplement: Supplementary file 1 [file DataSheet_1.zip › Supplemental Material and Figures 1-4.DOCX]

**Supplementary Methods:**

**Bile acid quantification**

For standardization purposes, a mixture containing 51 unconjugated, taurine- and glycine-conjugated BAs and 16 BA sulfates and glucuronides was dissolved in 40% acetonitrile at 10 nmol/mL, and serial dilutions were made to give standard solutions with a range of concentrations. Each standard was mixed 1:1 with an internal standard solution containing 14 deuterium (D)-labelled BAs before being injected for UPLC-MS. Linear-regression calibration curves were generated from analyte-to-internal standard peak area ratios versus molar concentrations. Concentrations of detected BAs in intestinal content and serum samples were calculated from the internal standard-calibration, linear-regression calibration curves of individual BAs prepared in duplicate.

Intestinal content samples were weighed prior to and following lyophilization to provide wet and dry sample weights. Moisture content in these samples was calculated as follows: 100% - the percentage difference between wet weight and dry weight of the samples. Each sample was combined with 25 μL/mg dry mass of 70% aqueous acetonitrile and two 3-mm metal beads. The samples were homogenized at a shaking frequency of 30 Hz for 1 minute three times on a MM 400 mill mixer, followed by sonication in a water bath for 2 minutes. Samples were centrifuged at 21,000 x *g* at 10˚C to clarify the supernatant, which was then diluted by a factor of 10 with 40% acetonitrile and mixed 1:1 with an internal standard solution before being injected for UPLC-MS.

For serum samples, 20 μL of serum was mixed with 40 μL of the internal standard solution containing 14 D-labeled bile acids and 40 μL of acetonitrile. Samples were vortexed for 30 seconds and sonicated for 2 minutes in a water bath. The sample tubes were centrifuged at 21,000 x *g* for 10 min at 5˚C. 80 μL of clear supernatant was extracted and combined with 920 μL of water. The mixture was loaded onto a 60mg/1mL Waters Oasis HLB cartridge, which was activated with 1 mL of methanol and subsequently reconditioned with 1 mL of water before use. Under positive pressure, the flow-through fraction was discarded. BAs were eluted with 800 μL of methanol. The collected fraction was dried under nitrogen and the residue was reconstituted in 64 μL of 40% acetonitrile before samples being injected for UPLC-MS.

No data points were excluded from inclusion or analysis.

## **Gene expression analysis**

Samples from different experimental groups were evenly distributed across plates used for qPCR analyses. Forward and reverse primers were used at a final concentration of 300 nM each. The final concentration of cDNA used for reactions was dependent on the results of primer optimization. Primer efficiency was determined using a serial dilution of pooled cDNA samples, and a primer efficiency in the range of 90 – 110% was considered acceptable.

Primers used were as follows:

*Gapdh*

F: 5’-ATGACATCAAGAAGGTGGTG-3’

R: 5’-CATACCAGGAAATGAGCTTG-3’

*Cyp7a1*

F: 5’-AGCAACTAAACAACCTGCCAGTACTA-3’

R: 5’-GTCCGGATATTCAAGGATGCA-3’

*Cyp8b1*

F: 5’-GGCTGGCTTCCTGAGCTTATT-3’

R: 5’-ACTTCCTGAACAGCTCATCGG-3

*Arkr1d1*

F: 5’-TGCACACCACCAAATATCCCT-3’

R: 5’-CTTCACTGCCACATAGGTCTTC-3’

*Cyp27a1*

F: 5’-GCCTCACCTATGGGATCTTCA-3’

R: 5’-TCAAAGCCTGACGCAGATG-3’

*Cyp2c70*

F: 5’-TGGGCTTTTGCTCCTGCTGAAG-3’

R: 5’-TCAGTGTACGGCATGTGGTTCC-3’

*Baat*

F: 5’-GGAAACCTGTTTAGTTCTCAGGC-3’

R: 5’-GTGGACCCCCATATAGTCTCC-3’

*Slc10a2*

F: 5’-TGGGTTTCTTCCTGGCTAGACT-3’

R: 5’- TGTTCTGCATTCCAGTTTCCAA-3’

*Slc51a*

F: 5’-TTGTGATCAACCGCATTTGT-3’

R: 5’-CTCCTCAAGCCTCCAGTGTC-3’

*Slc51b*

F: 5’-GTATTTTCGTGCAGAAGATGCG-3’

R: 5’-TTTCTGTTTGCCAGGATGCTC-3’

*Fgf15*

F: 5’-GCCATCAAGGACGTCAGCA-3’

R: 5’-CTTCCTCCGAGTAGCGAATCAG-3’

*Nr1h4*

F: 5’-TCCAGGGTTTCAGACACTGG-3’

R: 5’-GCCGAACGAAGAAACATGG-3’

*Gpbar1*

F: 5’-AAAGGTGTCTACGAGTGCTTC-3’

R: 5’-TGCATTGGCTACTGGTGTG-3’

*Nrb02*

F: 5’-CGATCCTCTTCAACCCAGATG-3’

R: 5’-AGGGCTCCAAGACTTCACACA-3’.

**Cycling conditions were as follows:**

| **Step** | **Temp (°C)** | **Time (seconds)** | **Repeated** |
| --- | --- | --- | --- |
| Preincubation | 50 | 120 | --- |
|  | 95 | 120 | --- |
| Denaturation | 95 | 15 | 45 cycles |
| Annealing | 60* | 15 |  |
| Extension | 72 | 60 |  |
| Melting | 95 | 15 | --- |
|  | 60 | 60 | --- |
|  | 95 | 1 | --- |

* This annealing temperature was used for amplification of all genes, with the exception of reactions to detect *Akr1d1*, *Cyp27a1*, *Fgf15*, *Nr1h4*, *Gpbar1*, and *Nr0b2*, where an annealing temperature of 55**°**C was used (all other parameters were unchanged).

## **Blinding**

Bile acid quantification was performed at the UVic Genome BC Proteomics Centre, Victoria, Canada, and technicians at this centre were not aware of experimental groups associated with sample identification numbers. Downstream analysis of bile acid concentrations and interpretation of data were performed predominantly by JML, TPB, and LAR, who were aware of experimental groups.

RNA isolations, gene expression analyses and data interpretation were performed predominantly by JML, TPB, and LAR, who were aware of experimental groups.

**Animal monitoring**

The approved animal use protocol governing all experimental work described in this manuscript includes set human clinical endpoints and monitoring of animals. Mice were checked at least daily throughout this study. Mice were monitored following helminth infection or mock infection to ensure no adverse clinical health outcomes resulting from the oral gavage infection procedure as well as resulting from the helminth infection. No animals in this study needed to be euthanized prior to the planned experimental endpoints.

**
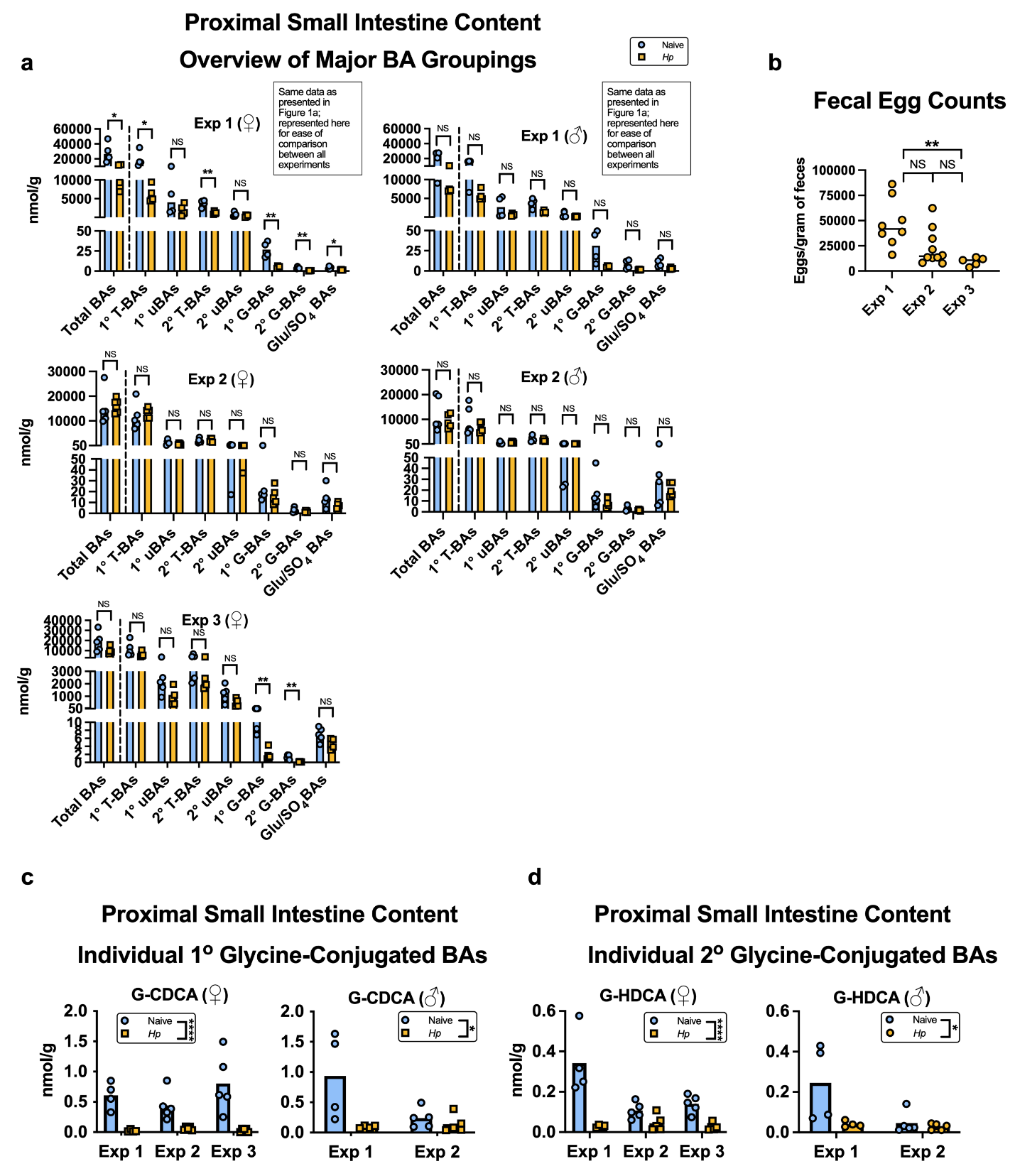
**

**Figure S1. Small intestinal *Heligmosomoides polygyrus* (*Hp*) infection disrupts bile acid (BA) homeostasis in the proximal small intestinal lumen.** **a.** For three independent experiment (Exp)s where mice were left naive or infected with different batches of *Hp* larvae, total BA concentrations are presented (left side of the dotted line), as well as concentrations of the major groupings of BAs (right side of the dotted line; primary [1°] T- [taurine-conjugated] BAs, 1° unconjugated [u] BAs, secondary [2°] T-BAs, 2° uBAs, 1° glycine-conjugated [G-) BAs, 2° G-BAs, and gluconated or sulfated [Glu/SO_4_] BAs). ‘Exp 1’ data is also presented in Figure 1a and is reproduced here for ease of comparison between experiments. Bile acid (BA) concentrations were normalized to wet sample weights. For Exp 1, *n*=4 naive female, *n*=4 infected female, *n*=4 naive male, and *n*=4 infected male. For Exp 2, *n*=5 naive female, *n*=5 infected female, *n*=5 naive male, and *n*=5 infected male. For Exp 3, *n*=5 naive female and *n*=5 infected female. Statistical comparisons between naive and infected mice for total BA levels as well as each groups of BAs were made using multiple t-tests (consistent standard deviations between samples not assumed, 5% false discovery rate (FDR), two-stage step-up method of Benjamini, Krieger, and Yekutieli). NS= not significant, * = q ≤ 0.05, ** = q ≤ 0.01. **b.** Egg counts per gram of feces across the independent experiments where mice were infected with different batches of infectious *Hp* larvae. Statistical comparisons were made using a Kruskal-Wallis test followed by a Dunn's multiple comparisons test. ** = p ≤ 0.01, NS= not significant. **c,d.** Concentrations of individual 1° G-BAs (**c**) and individual 2° G-BAs (**d**) across independent experiments, each with an *n*=4-5 in each experimental group. Two-way ANOVA tests were used to assess how Exp and infection status contributed to variation within data sets and the results of the impact of infection status are presented on graphs. * = p ≤ 0.05, **** = p ≤ 0.0001. Each point represents data from an individual mouse.

**
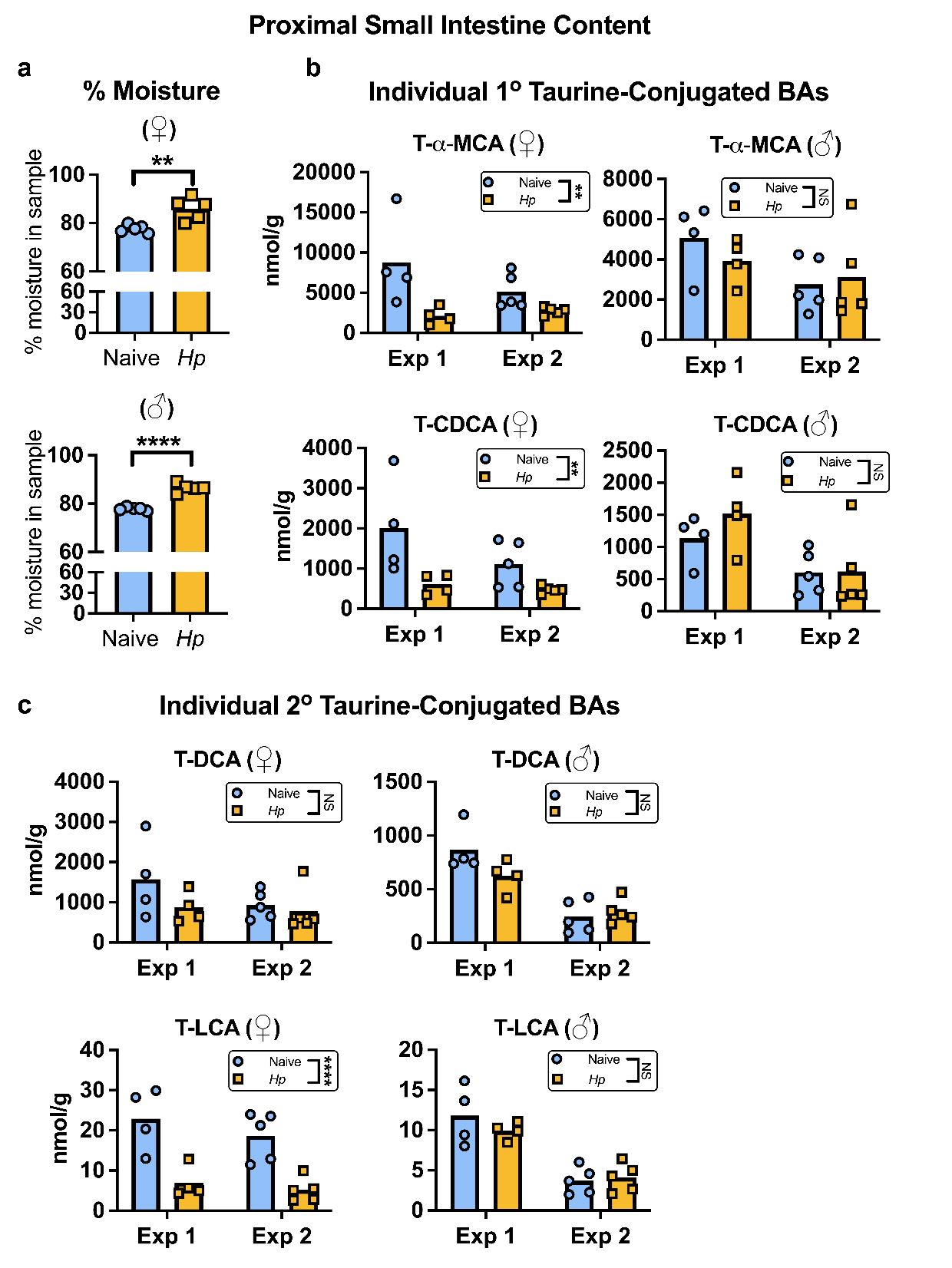
**

**Figure S2. Disruption to bile acid (BA) concentrations in the proximal small intestine may be partially due increased luminal fluid during *Heligmosomoides polygyrus* (*Hp*) infection.** Female (♀) and male (♂) mice were left naive or infected with *Hp*. Small intestinal moisture content was calculated and BA concentrations in the proximal small intestinal contents were normalized to dry sample weights. Each data point represents results from an individual mouse. **a.** Percent moisture in proximal small intestinal content samples. Data shown are from one experiment and are representative of results from two independent experiments, each with an *n*=4-5 in each experimental group. Statistical comparisons were made using an unpaired t-test. **b,c.** Concentrations of individual primary (1°) taurine-conjugated (T-) BAs (**b**) and individual secondary (2°) T-BAs (**c**) across independent experiment (Exp)s, each with an *n*=4-5 in each experimental group. Two-way ANOVA tests were used to assess how Exp and infection status contributed to variation within data sets and the results of the impact of infection status are presented on graphs. NS= not significant, ** = p ≤ 0.01, **** = p ≤ 0.0001. Each point represents data from an individual mouse.

**
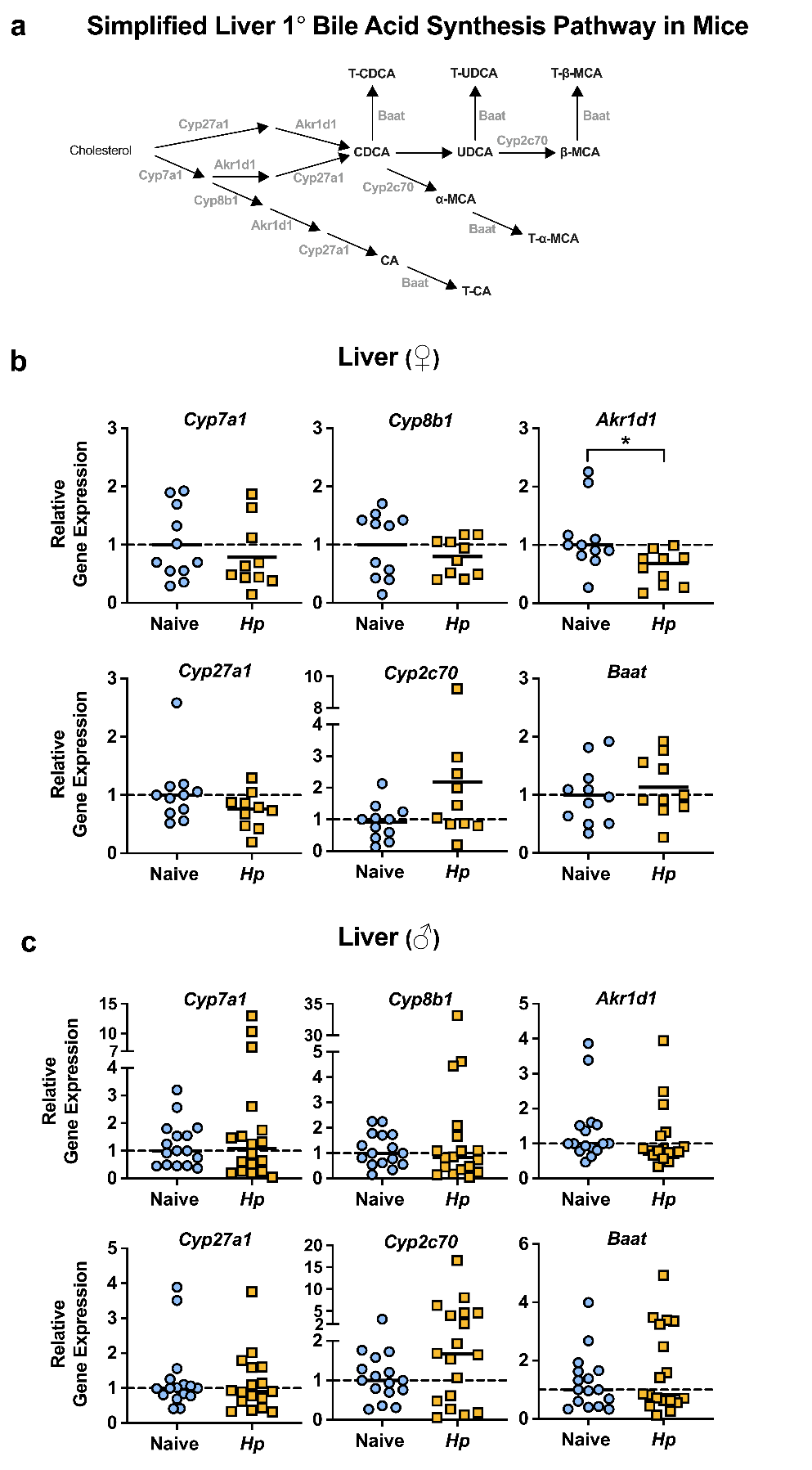
**

**Figure S3. *Heligmosomoides polygyrus (Hp)* infection results in decreased expression of *Akr1d1* in the livers of female mice. a.** Simplified schematic of the primary (1°) bile acid (BA) synthesis pathway in mice (Li and Dawson, 2019; Gathercole et al., 2022). Note that several intermediate steps in synthesis reactions are not shown in this schematic for simplicity. Data shown for ♀ mice (**b**) are pooled from two independent experiments, each with an *n*=4-6 in each experimental group (combined *n*=11 naive and *n*=10 infected mice), and data shown for ♂ mice (**c**) are pooled from three independent experiments, each with an *n*=5-6 in each experimental group (combined *n*=16 naive and *n*=18 infected mice). Statistical comparisons were made using unpaired t-tests if data were parametric, or Mann-Whitney tests if data were non-parametric. * = p ≤ 0.05. Each point represents data from an individual mouse.

**
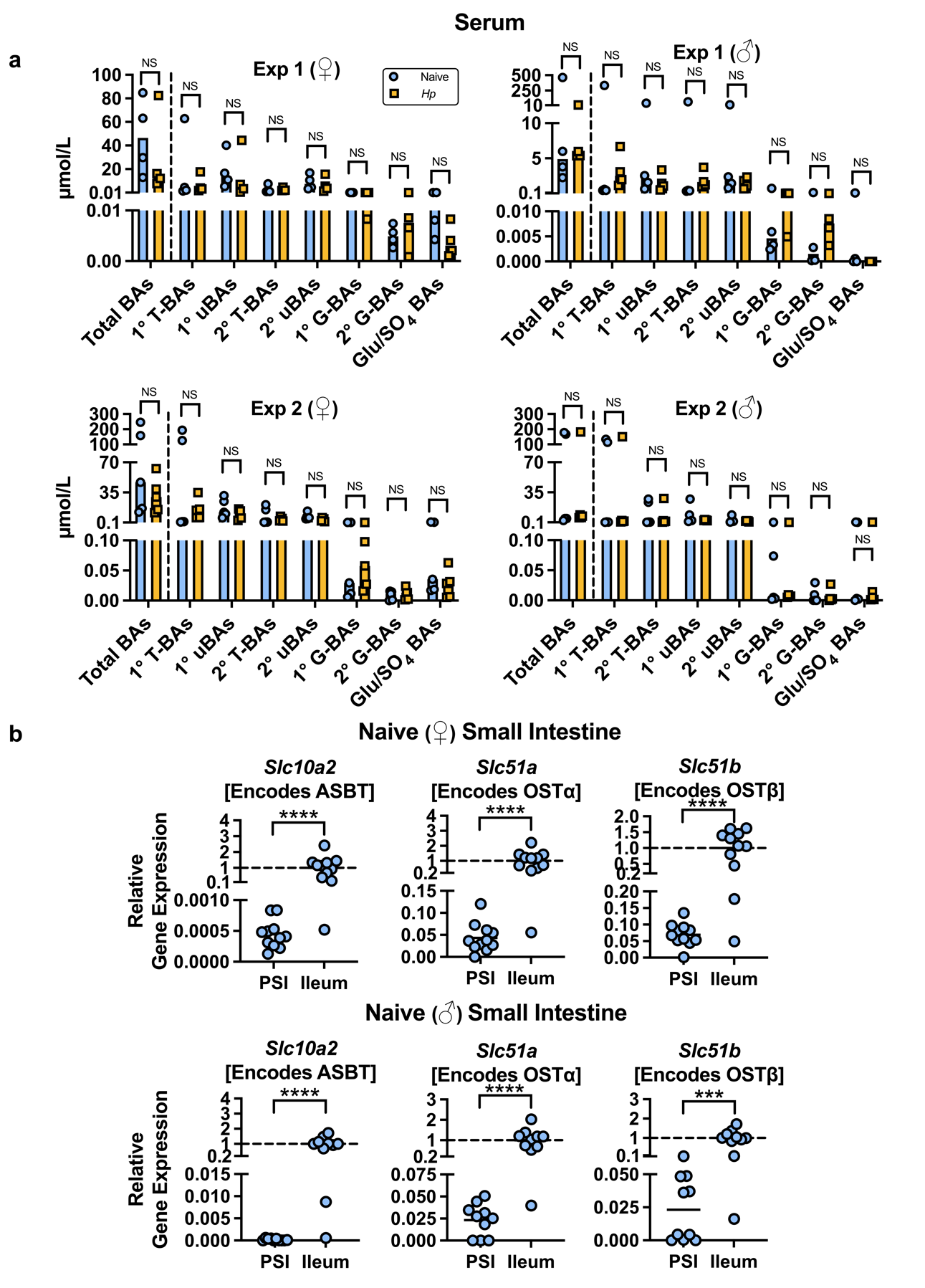
**

**Figure S4. Small intestinal *Heligmosomoides polygyrus* (*Hp*) infection does not impact serum bile acid (BA) concentrations.** **a.** For two independent experiment (Exp)s where mice were left naive or infected with different batches of *Hp* larvae, total BA concentrations are presented (left side of the dotted line), as well as concentrations of the major groupings of BAs (right side of the dotted line; primary [1°] T- [taurine-conjugated] BAs, 1° unconjugated [u] BAs, secondary [2°] T-BAs, 2° uBAs, 1° glycine-conjugated [G-] BAs, 2° G-BAs, and gluconated or sulfated [Glu/SO_4_] BAs). For Exp 1, *n=*4 naive females, *n*=4 infected females, *n*=4 naive males, and *n*=4 infected males. For Exp 2, *n=*5 naive females, *n*=5 infected females, *n*=5 naive males, and *n*=5 infected males. Statistical comparisons between naïve and infected mice for total BA levels as well as each groups of BAs were made using multiple t-tests (consistent standard deviations between samples not assumed, 5% false discovery rate (FDR), two-stage step-up method of Benjamini, Krieger, and Yekutieli). **b.** Expression levels of the indicated BA transporter genes were determined in the proximal small intestinal tissual and ileal tissue by quantitative PCR. Data shown are pooled from two independent experiments, each with an *n*=5-6 in each experimental group (combined *n*=10 naive females, *n*=10 infected females, *n*=11 naive males, and *n=*11 infected males). Statistical comparisons were made using unpaired t-tests if data were parametric, or Mann-Whitney tests if data were non-parametric. *** = p ≤ 0.001, **** = p ≤ 0.0001.

**References**

Gathercole, L. L., Nikolaou, N., Harris, S. E., Arvaniti, A., Poolman, T. M., Hazlehurst, J. M., et al. (2022). AKR1D1 knockout mice develop a sex-dependent metabolic phenotype. *J. Endocrinol.* 253, 97–113.

Li, J., and Dawson, P. A. (2019). Animal models to study bile acid metabolism. *Biochim. Biophys. Acta - Mol. Basis Dis.* 1865, 895–911.
